# Markers of cognitive skills important for team leaders in emergency medical services: a qualitative interview study

**DOI:** 10.1186/s12873-022-00629-1

**Published:** 2022-05-06

**Authors:** Martin Sedlár, Zuzana Kaššaiová

**Affiliations:** Institute of Experimental Psychology, Centre of Social and Psychological Sciences, Slovak Academy of Sciences, Dúbravská cesta 9, 841 01 Bratislava, Slovakia

**Keywords:** Non-technical skills, Situation awareness, Decision making, Prehospital, Cognitive work analysis

## Abstract

**Background:**

Cognitive skills and other non-technical skills are key for emergency medical services (EMS); however, there have been a limited number of their markers identified. This study aims to identify markers of cognitive skills—situation awareness and decision making—important for team leaders in EMS. The focus is on any markers of cognitive skills that are associated with quality and safety at EMS work.

**Method:**

In-depth semi-structured interviews were conducted with 20 EMS team leaders (10 EMS physician team leaders and 10 paramedic team leaders) and analysed by the structured approach utilising the known framework of cognitive skill elements.

**Results:**

The data analysis revealed a set of 50 markers falling into elements of situation awareness (gathering information, interpreting information, anticipating states), elements of decision making (identifying options, implementing decisions, re-evaluating decisions), and an additional cognitive element (maintaining standards). These markers represented cognitive processes, acts, and communications, therefore, some of them can be observable and others rather unobservable. The identified markers were not too specific, applicable mostly in various challenging situations with patients’ medical problems and in EMS team leaders working in ground ambulances in urban and rural/remote areas.

**Conclusion:**

The findings provide a better understanding of EMS team leaders’ cognitive skills, and can aid in the development of assessment and training tools suited particularly to them.

**Supplementary Information:**

The online version contains supplementary material available at 10.1186/s12873-022-00629-1.

## Background

Decision making, simply defined as choosing an option to implement, is based on situation awareness, described as being aware of what a situation is about [1, 2]. These two interconnected cognitive skills belong to key non-technical skills, complementing medical and technical skills, for emergency medical services (EMS) professionals [3–5]. They contribute to a lower incidence of unsafe behaviour and EMS-patient and -personnel safety incidents [6]. In other words, better and safer performance could be achieved if these skills were improved to a higher level [2, 7].

The general cognitive skill categories can be further divided into less general elements and specific markers. Markers are aspects of performance indicating the presence or absence of a requisite cognitive or behavioural process [8]. Ultimately, markers that can be observed are what the concept of non-technical skills, with its interest in developing observer-assessment and training tools, is all about. However, despite attempts to find such markers, some are only indirectly observable when externalised in acts and communications [9], and others may be even unobservable. To our knowledge, there have been three published studies covering observable behavioural markers of cognitive skills for the EMS occupation [10–12]. More studies have described essential, though not necessarily observable, attributes [3, 5, 13]. In sum, though there has been an increasing tendency to examine cognitive skills in EMS, a limited number of their associated markers potentially applicable to various EMS professionals have been identified [4].

Cognitive skills are especially important for team leaders in EMS. Leaders usually have extensive education and/or experience, and they possess the biggest burden of responsibility [14]: they are the main decision-makers who should make correct, safe, and efficient decisions based on accurate situation awareness. This does not mean that other team members do not use cognitive skills. They usually need to be situationally aware, but do not make crucial decisions [15]. Rather, they are followers of EMS leaders’ directions. Markers should be defined for each team member to make clear recommendations for practice, but so far few studies on non-technical skills have explicitly addressed this issue in emergency settings [15–18].

Generally, cognitive skills are ascribed to individual cognition. In this context, this refers to EMS team members’ minds. However, to function effectively, EMS team members need to interact with one another through team processes, what is called team cognition [8], and they also need to interact on and off a scene with other medical and non-medical professionals, utilising standards and technology, which represents distributed cognition [19]. In addition, a certain degree of shared understanding or shared decision making in relation to a patient, his/her relatives, or significant others is of great value [20, 21]; however, including this aspect of cognition in non-technical skills taxonomies is scarce [3, 22].

This study aims to identify markers of cognitive skills—situation awareness and decision making—important for team leaders in EMS. The focus is on any markers of cognitive skills that could guarantee the quality and safety of EMS work and be used for developing assessment and training tools to enhance performance.

## Method

### Design

A qualitative research design with individual in-depth semi-structured interviews about cognitive skills important for EMS team leaders was used. It was informed by the naturalistic decision-making paradigm and the concept of non-technical skills.

### Setting

This study was carried out in Slovakia, where there are two basic types of EMS ground-ambulance teams. Physician-led teams have three members: a physician, a paramedic, and a driver/paramedic with an ambulance driving licence. Paramedic-led teams have two members: a paramedic and a driver/paramedic with an ambulance driving licence. In physician-led teams, the formal leader is the physician by default, due to their more advanced education. In paramedic-led teams, the formal leader is usually the one who has paramedical education and is not driving an ambulance during a shift. Slovak EMS teams usually provide prehospital emergency care in urban and rural/remote areas depending on the call and the place of the EMS station.

### Participants and recruitment

The research sample consisted of 20 participants (Table 1). One more participant—the first to be interviewed and serving as a guide for trialling and refining an interview schedule—was not included in the sample. All potential participants were recruited through email requests sent to companies providing EMS, requests posted on specialised social network groups, or direct email contact with previous participants. They were offered a €30 gift voucher per interview. From a list of registered potential participants, those for this study were purposefully selected according to criteria of equal numbers of both sexes, only team leaders working in ground ambulances, equal numbers of both professions, i.e., EMS physician and paramedic team leaders, and only experienced team leaders, as opposed to novices. The lower limit for taking part in the study was set at 3 years of working in EMS. This limit was based on Benner’s novice to expert model—two to 3 years of clinical work experience are needed to become a competent performer [23]. Registered interested participants who met the criteria were contacted via email. There was no prior interaction or relationship between the authors of this study and its participants, except for two, who had been interviewed once before by the first author for a previous study.

**Table 1:** Characteristics of the participants

Total	20
Sex	
Male	10
Female	10
Profession	
EMS physician	10
Paramedic	10
Age, years	
Range	30–63
Mean (Standard deviation)	44.45 (8.66)
Work experience in emergency medical services, years	
Range	3.5–30
Mean (Standard deviation)	16.05 (8.09)
Work experience in health care, years	
Range	3.5–37
Mean (Standard deviation)	20.63 (9.11)

### Interview schedule and data collection

Data were collected between August and December 2019 by the first author, who has acquired experience in interviewing EMS professionals while working on previous studies and research projects. Upon agreement, each individual interview was conducted in person at a convenient time and place free from distractions and other people. At the beginning of the meeting, participants were informed about the research and its conditions, and provided written informed consent. After asking basic questions about the participant and job, the interview followed a two-part in-depth semi-structured interview schedule. Both parts were based on the adaptation of critical incident methods [24, 25], utilising the known framework of non-technical cognitive skill elements (see Table 2) [2, 4] and eliciting markers of these elements. In the first part, participants were asked to choose one specific cognitively challenging situation from their practice in EMS—a situation in which they led a team and used their expert cognitive skills. Then, they were asked deepening probe questions about cognitive skill elements. In the second part, they were asked to make generalisations concerning EMS leaders’ cognitive skill elements and differences in routine and challenging situations. These two complementary parts were used to increase the comprehensiveness of the findings. In both parts, participants were guided not to think about mass-casualty situations, due to their unique nature and high demands on management skills. The interviews lasted 90–200 min, and were digitally audio-recorded. Finally, a €30 gift voucher was given to each participant.

**Table 2:** Definitions of cognitive skill elements

Skills	Elements	Definitions
Situation awareness	Gathering information	Continually collecting available important information about a situation to have updated information
	Interpreting information	Interpreting information gathered to have an updated mental model of a situation
	Anticipating states	Anticipating what may happen in the near future, what outcomes and consequences will be probable in a situation after applying an intervention or non-intervention
Decision making	Identifying options	Identifying several possible alternatives for solving a situation and considering the positives and negatives of each
	Implementing decisions	Choosing and implementing a course of action and communicating it to others
	Re-evalauting decisions	Continually re-evaluating an implemented course of action and changing it when needed
	Maintaining standards	Engaging in formal and informal quality and safety practices in work that support cognitive skills

### Data analysis

Data analysis of the whole verbatim and anonymised transcriptions of interviews was carried out using the software Atlas.ti 7. It was based on the structured approach originally developed for the analysis of data from the critical decision method [26]. This approach uses an a priori framework for identifying items of interest. In our study, the a priori framework was represented by the known structure of cognitive skill elements suitable for the EMS setting (see Table 2) [2, 4]. Items of interest were represented by the markers. The whole data analysis process had several steps. At first, reading transcriptions and creating short summaries of the challenging situations served as familiarisation. Next, each statement indicating a marker relevant to one of the cognitive skill elements was identified. Then, similar statements were grouped together and meaningfully and aptly labelled. During the analysis, identifying, grouping, and labelling statements tended to be performed simultaneously, which was facilitated by the software. In this way, the first author created an initial version of the codebook, i.e., an initial list of markers. Then, the second author got the initial version of the codebook and analysed the data independently. Finally, the analysers’ coding was compared and disagreements were resolved by discussions until reaching consensus on a final version of the codebook, i.e., a final list of markers.

## Results

Overall, 50 markers of cognitive skills were identified belonging to the predefined elements of cognitive skills. Participants described principally markers of cognitive skills associated with patients’ medical and physical problems. The summary of the main findings—the identified markers—is displayed in Table 3. They are described in more detail in the following sections, ordered according to cognitive skill elements introduced in Table 2. Representative examples of participants’ statements are in the Supplementary Table.

**Table 3:** Markers of cognitive skills important for team leaders in emergency medical services

Skills	Elements	Markers
Situation awareness	Gathering information	Conducts an initial scan of a situation
		Assesses a patient
		Monitors a patient
		Observes actions of team members and other professionals
		Cross-checks information
		Discusses with or considers suggestions from team members regarding what information to gather
		Communicates information about a situation to team members
		Involves team members in gathering information
		Eliminates distractions
		Gathers comprehensive information systematically
		Adapts gathering information to relevant and priority information
	Interpreting information	Demonstrates understanding of a patient’s condition and its changes
		Makes a working diagnosis
		Generates and considers an adequate number of different diagnoses
		Identifies and resolves inconsistencies
		Communicates interpretations regarding a patient’s condition and diagnosis to team members
		Discusses with or considers suggestions from team members regarding a patient’s condition and diagnosis
	Anticipating states	Anticipates a possible course of events
		Prepares for a possible negative course of events with alternative plans
		Takes action for the sake of a possible negative course of events
		Communicates a possible negative course of events to team members
		Discusses with or considers suggestions from team members regarding a possible negative course of events
		Involves team members in anticipation-related activities
Decision making	Identifying options	Generates and considers an adequate number of alternative solutions
		Discusses with or considers suggestions from team members regarding solutions
		Considers advantages and disadvantages of solutions
		Evaluates relevant factors and is not affected by irrelevant factors
		Seeks input on various situation-related issues with relevant parties
		Involves team members in seeking input with relevant parties
		Identifies leverage points and uses them in devising novel solutions
	Implementing decisions	Implements a solution while gathering information
		Prepares tasks before implementing
		Tailors workspace and bystanders’ behaviour
		Involves team members and other relevant resources in solving a situation
		Communicates and explains decisions and actions to team members, other professionals, patients, and their close ones
		Provides adequate situation reports
		Implements a comprehensive solution systematically
		Adapts implementing a solution to relevant and priority actions in a situation
	Re-evaluating decisions	Re-assesses a patient
		Adapts re-assessing a patient to relevant and priority information
		Allows adequate time for intervention to take effect
		Revises a solution in light of new information
		Searches for more information and other options
		Involves team members in re-assessing a patient
	Maintaining standards	Follows established practice guidance when appropriate
		Can justify when not following established practice guidance
		Leads team members to follow established practice guidance
		Uses relevant experience and knowledge
		Shows professional behaviour
		Engages in learning activities

### Gathering information

Leaders emphasised conducting an initial scan of a situation to identify its seriousness and critical points, assessing a patient to identify his/her problems/needs, monitoring a patient, and cross-checking information to increase reliability. For these purposes, leaders had to collect information using multiple means and from multiple sources. To gain the big picture of a situation, they also had to observe team members and other professionals, including their actions performed in the interest of a patient.

Leaders also pointed out discussing or considering spontaneous suggestions from team members regarding what other information would be good to gather and communicating information about a situation to team members when beneficial. Further, involving team members in tasks associated with gathering information was important. Sometimes, there was also a need to eliminate distractions, e.g., disruptive bystanders’ communications or loud music, to support gathering information. Two more markers are significant here: gathering comprehensive information systematically within available possibilities of EMS practice, and adapting such information gathering to the most important information with respect to relevance and priority.

### Interpreting information

One of the identified markers was to demonstrate understanding of a patient’s condition and its changes. A common practice was also to make a working diagnosis. Even though experienced leaders could rapidly recognise what a patient’s problem was, generating and considering an adequate number of relevant alternative diagnoses represented a safer approach. This way, they could avoid fixating on the first or expected interpretation that popped into their mind, which might not necessarily be correct.

From time to time, leaders identified inconsistencies, i.e., cases where some information did not fit into the actual mental model, and resolved them, which resulted in changing or updating their mental model. According to them, it was useful to verbalise their interpretations, such as a patient’s condition and its changes, diagnostic thoughts, and reasoning behind their interpretations. These verbalisations enhanced shared understanding and enabled other team members to learn, when they had less experience and knowledge, and eventually correct team leaders’ interpretations. When needed, they even initiated discussions and considered team members’ spontaneous suggestions and thoughts in this regard.

### Anticipating states

For leaders, an important marker of this element was being able to anticipate a possible course of events in itself. In serious cases, they anticipated bad or the worst possibilities, i.e., deteriorations in a patient’s condition or complications in a situation. Only after the seriousness had disappeared would they think about a positive course of events. While anticipating negative possibilities, leaders highlighted the significance of a proactive approach: to be prepared by creating an alternative plan B or even plan C and to take action in advance to avoid or mitigate problems.

The leaders stressed informing their team members about a possible negative course of events to make them sensitive to noting relevant cues and not to be surprised. Quite useful was also to initiate a discussion about a possible negative course of events with team members. The last marker in this element was to involve team members in preparing for and taking action to avoid or mitigate a possible negative course of events. This meant to instruct on or take for granted tasks associated with the proactive approach.

### Identifying options

Although leaders were able to recognise an adequate option and implement it rapidly, some challenging situations required them to generate and consider alternative courses of action to arrive at the proper one. Therefore, they also initiated discussions about solutions with team members and considered their spontaneous suggestions. This and any mentioned kind of discussions with team members were praised and carried out mainly when leaders were less experienced, more uncertain, and when they could rely on team members’ knowledge and experience. However, some leaders noted that discussions could be perceived by patients and bystanders as a sign of EMS-team incompetence and potentially be followed by complaints. That is why they preferred discussions when a patient is unconscious, when a patient’s relatives and significant others are not present, or by speaking in codes and hints that team members understand, unlike laypeople.

Leaders usually considered advantages and disadvantages of solutions respecting a patient and a situation. It was also important to evaluate relevant factors, e.g., time criticality, distance, resources, or technical issues, and not to be inappropriately affected by irrelevant factors, e.g., a patient’s social status. When needed, leaders themselves or by involving team members sought input on various situation-related issues with relevant parties. Because of the impossibility of being prepared for every single task in an unpredictable situation, leaders also emphasised improvisation—identifying leverage points, such as tools or materials seen on a scene, and using them in devising novel solutions.

### Implementing decisions

Challenging situations frequently required leaders to implement a solution while gathering information or at least to prepare certain tasks before implementing them. Both these behaviours helped them to shorten the time taken. Certain cases called for tailoring inappropriate workspaces and bystanders’ behaviour to support implementing solutions, e.g., to relocate a patient or objects, to place EMS bags nearby, or to keep bystanders busy with something so as not to be too intrusive. Further, leaders stated the necessity of involving mainly team members in implementing a solution. When needed, they also involved other professionals and bystanders present on a scene in solving a situation.

It was identified as beneficial to enhance shared understanding by informing patients, their close ones, team members, and other professionals about decisions and actions to know what is going to be done and eventually to explain reasons for such decisions and actions. Providing adequate situation reports to another EMS team or hospital staff while handing over a patient served for subsequent health care. Other identified markers were: implementing a comprehensive solution systematically with available treatment and transport options, and adapting such a solution to the most important actions regarding relevance and priority.

### Re-evaluating decisions

Re-assessing a patient was performed after an intervention, but also after some time without any intervention just to check the patient. As leaders noted, it was effective to adapt re-assessing a patient to the most important information regarding priority and relevance. They further acknowledged allowing adequate time for interventions to take effect and to revise a course of action when new information becomes apparent, regardless of an intervention or non-intervention.

When a patient’s condition was not getting better or was getting worse, to search for more information and other options was regarded as essential behaviour. Leaders also involved team members in re-assessing a patient when needed. In this and previous markers labelled as involving, there were identified two ways of involving team members—either instructing them what to do or taking their tasks for granted. They usually instructed them when it was an individual preference of the leader or the team member, when working with a new/unfamiliar team member, when encountering a challenging situation, or when they could not fully rely on team members to know what they were supposed to do.

### Maintaining standards

Following established adequate practice guidance when appropriate was illustrated with many examples of good and safe practice. When some of the key established practice guidance had not been followed by the leader, being able to justify (to a superior after a situation) why that had happened was crucial. When team members had done that, the leader told them (immediately in a situation or while debriefing) to follow established practice guidance.

To use experience and knowledge was also stated as necessary for cognitive skills and usually interconnected with applying practice guidance. While working, leaders deemed it necessary to show professional behaviour towards patients, bystanders, and team members. This meant being respectful, courteous, interested in welfare, calm, and acting decisively and based on awareness of one’s own limits. Moreover, they praised engaging in various learning activities, e.g., participating in simulation trainings or obtaining feedback.

## Discussion

The study aimed to investigate markers of cognitive skills—situation awareness and decision making—important for team leaders in EMS. The analysis revealed 50 markers that fell under seven elements: gathering information, interpreting information, anticipating states, identifying options, implementing decisions, re-evaluating decisions, and maintaining standards. Each marker was associated with quality and safety at work.

### Novelty of markers

The novelty lies in incorporating the additional cognitive element, i.e., maintaining standards and its markers, as an integral part of cognitive skills [4]. Moreover, the analysis suggests that some identified markers of cognitive skills are associated with prioritising, planning, and preparing—it is not surprising since these processes are basically cognitive. A similar approach incorporating these aspects into cognitive skills can be found in previous studies [10, 12], and the approach of separating them from cognitive skills can be found too [11, 27]. The results also yielded markers associated with communication and coordination. We included only those tightly intertwined with cognitive skills—specifics about what communication and coordination should look like were excluded as relevant for social skills.

In the data analysis, our attention was not narrowed to only markers related to the cognition of leaders, their teams, and other necessary elements of the whole sociotechnical system [19]. This is a typical approach in non-technical skills research, though not declared explicitly. In accordance with other studies [3, 20, 22], we also embraced the importance of cognition or shared understanding between leaders on one side and patients, their relatives, or significant others on the other.

All things considered, we have identified a new set of markers; however, it can be claimed that some of the markers are similar, at least in terms of label applied, to previous research in prehospital [10, 11] or hospital settings [27, 28]. This is quite understandable, due to similarities and differences in study designs and across examined settings.

### Specificity of markers

In our data analysis and in reporting the results, we proceeded from general categories through less general elements to specific markers. This three-level system of genericity/specificity is common in non-technical skills research, since its purpose is to be applicable in most situations relevant to an occupation. While the categories are usually widely valid, elements and markers vary among occupations/work settings [2]. Thomas [7] pointed out that the usual level of specificity of behavioural markers is limited, and much more powerful assessment tools without difficulties in interpreting such generic behaviours in a specific situation could be created based on a highly specific set of behavioural markers. Of course, that would require creating separate highly specific sets of markers for each individual situation. Another option could be to create at least specific sets of markers for prototypical situations, e.g., for cardiac arrest resuscitation [18]. Anyway, a review has concluded that in contrast to tools designed for assessing very specific actions (checklists), tools designed for assessing overall technical and non-technical performance at a general level (global rating scales) have higher reliability, can be used across multiple tasks, and may better capture nuanced elements of expertise [29].

### Observability of markers

We identified several behavioural markers that are probably observable (e.g., communicating interpretations to team members or re-assessing a patient). The issue here arises when it comes to construct validity. Can cognitive skills that are cognitive in their essence be observed and assessed accurately from outside? Scholars answer that although cognitive skills cannot be directly observable, at least they can be inferred from associated actions and communications [2, 9]. The EMS profession is distinguished by many actions and communications with multiple people on and off a scene. Nevertheless, there is still a certain degree of doubt regarding how much of the external behaviour offers an insight into cognitive skills [30] and to what extent subtle behaviour is easily noticeable [27].

Furthermore, we also identified markers that may be rather unobservable (e.g., anticipating a possible course of events or considering advantages and disadvantages of solutions). They represent internal mental activities deemed to be important for EMS according to participants. In contrast with the principle of excluding them from observer-based assessment tools [9], we propose assessing markers and cognitive skills in general by multiple methods. Besides the mentioned, there are alternative methods that are based on the subjective assessment of own skills and the administration of skill-related queries during or after a simulation of a task. As each of the methods has multiple pros and cons [30], combining methods can increase the reliability and validity of assessments.

### Applicability of markers

When it comes to settings, our findings are applicable to EMS team leaders working in ground ambulances in urban and rural/remote areas in Slovakia. The comparison of these findings with findings from diverse prehospital and hospital settings in other countries [10, 11, 27, 28] implies partial relevance or overlapping of the identified markers also with other settings.

During the interviews, participants applied their descriptions particularly to patients’ medical or physical problems, which was stimulated by the interview schedule itself, accentuating cognitive difficulty. Therefore, the markers identified here may apply more to such situations than routine ones. Additionally, participants suggested that besides situational factors, leaders’ behaviours towards other team members depend also on team factors, such as familiarity, experience, knowledge, uncertainty, or preferences of team members. Particular behaviours towards them can be viewed as examples of explicit and implicit coordination [31].

This study focused on the leaders’ cognitive skills, and thus the applicability of these markers is self-evident. Whether these markers can also be applied to the followers’ cognitive skills is questionable. However, occasional switches between team roles [32, 33] indicate that there could be cognitive skills’ markers from our results appropriate to both leaders and followers in EMS teams.

Our research sample consisted of EMS physician leaders and paramedic leaders. The findings apply to both—this not surprising for three reasons. First, they talked about the same markers of non-technical cognitive skills. Second, in Slovakia, they work in the same prehospital emergency setting and encounter more or less similar situations, although physician-led teams are usually delegated to more serious cases than paramedic-led teams. Third, non-technical skills are conceptualised as general skills, so their applicability is wider. However, the two kinds of professionals likely differed in medical and technical skills, conceptualised as specific skills, analysis of which was beyond our scope.

From all these points, it follows that the applicability of all markers under any circumstances has its limits, and hence further scrutiny is vital. This issue has already been pointed to in previous research on markers, e.g., by incorporating a “not applicable” option in the assessment tools [10, 11].

### Limitations

There are six potential limitations. First, our results apply to the Slovak EMS setting, so their applicability to EMS settings in other countries should be viewed with caution. Second, our study provides findings on markers of cognitive skills that are important from EMS team leaders’ perspective, therefore, we cannot comment on which markers of cognitive skills other team members, expert panels, or patients consider important. Third, we cannot say for sure which of the identified markers are definitely observable or unobservable, since this was not the study’s aim. Fourth, our results can be limited due to individual participants’ ability to accurately recollect specifics about past situations and to make generalisations, as required by the two-part interview schedule. Fifth, although the first author’s greater knowledge base about cognitive skills in EMS helped in navigating the whole research process, alongside this there was a risk of bias due to possible preconceptions. However, the second author, who was less knowledgeable in the topic of the research, functioned as a counterbalance. Sixth, findings from qualitative research may be incorrectly regarded as lacking generalisability. In fact, they are conceptually generalisable, indicating that the identified markers can be used for understanding cognitive skills in emergency medicine in general.

## Conclusion

Just as in other high-stakes, dynamic, and complex settings, cognitive skills are essential for EMS. This study looked closely at them, aiming to identify markers important exclusively for team leaders in EMS. A new set of markers emerged within two cognitive skill categories—situation awareness and decision making—and their elements. These markers include examples of team leaders’ cognitive processes, their acts, and communications, thus, some of the markers can be observable and others rather unobservable. The adequate level of the markers’ specificity predetermines their applicability to various situations, but principally to challenging emergency situations with patients’ medical and physical problems. They are relevant for such EMS team leaders who are either physician leaders or paramedic leaders working in ground ambulances in urban and rural/remote areas. Overall, our findings contribute to a better understanding of what is required from EMS team leaders, and lay a solid foundation for the development of assessment and training tools focused on cognitive skills. Consequently, these findings may play a pivotal role in improving the safety and quality of EMS work.

## Supplementary Information

**Additional file 1.**

## Data Availability

The datasets generated and analysed during the current study are not publicly available since this would compromise the informed consent signed by the participants. The informed consent contains a statement that only research-project members would have access to the raw data and that findings would be presented in an anonymised way.

## Abbreviations

EMS: Emergency Medical Services
