# Supplementary material for: Markers of cognitive skills important for team leaders in emergency medical services: a qualitative interview study
Source: BMC Emerg Med. 2022 May 6;22:80. doi: 10.1186/s12873-022-00629-1 (PMC9074215; doi:10.1186/s12873-022-00629-1)
Supplement: Supplementary file 1 — Additional file 1. [file 12873_2022_629_MOESM1_ESM.docx]

**Supplemental Table**

*Representative examples of participants’ statements*

| Elements | Markers | Statements |
| --- | --- | --- |
| Gathering information | Conducts an initial scan of a situation | *Automatically, subconsciously I started taking note of people lying somewhere. At the same time, I started counting how many people were there and how they were moving . . . Such a quick scan.* (P8) |
|  | Assesses a patient | *A good leader has to ask a lot. That is the basis. He or she has to notice the patient, environment, and surroundings, because many times even the surroundings can suggest some things, to observe reactions of relatives.* (P17) |
|  | Monitors a patient | *We had to continually monitor his level of consciousness, his pulse, his breath frequency, his oxygenation. That was provided by an ECG monitor and a fingertip oximeter.* (P15) |
|  | Observes actions of team members and other professionals | *I see peripherally what they’re doing. It is not like that I have my back to them and when I turn, I’m surprised what they are doing. For example, while I’m asking, they are placing a 12-lead ECG.* (P20) |
|  | Cross-checks information | *Sometimes there is a need to evaluate whether the patient is telling the truth. Some people just want to exploit the system, exploit the emergency services . . . It can be seen in their gestures and speech when they are lying. You can repeat questions from different perspectives later or ask the same thing of bystanders, relatives, and so on.* (P13) |
|  | Discusses with or considers suggestions from team members regarding what information to gather | *We are two paramedics and we can rely on each other, such that if I accidentally overlook something, my colleague will say, “Listen to me, we need this.” Even though I lead, the other one is there, I know he perceives it, listens, and he can intervene, and remind me of something.* (P2) |
|  | Communicates information about a situation to team members | *Actually, from the role of a paramedic, I say what is in the call, how it was notified.* (P18) |
|  | Involves team members in gathering information | *While I’m talking to a patient and taking history, the paramedic puts on ECG electrodes and a blood-pressure cuff. How he does the work within emergency services is logical, because when I am doing that, he is doing something else to obtain information. And we work as a team.* (P19) |
|  | Eliminates distractions | *I gave the order to let the music stop because it was still playing as if nothing had happened, which can be sometimes annoying.* (P14) |
|  | Gathers comprehensive information systematically | *Maybe comprehensiveness of information gathering. Whether the entire history is taken. This means taking personal, drug, allergic, social, and work history and so on, and in women also gynaecological information. Not to skip anything.* (P13) |
|  | Adapts gathering information to relevant and priority information | *In the more challenging situations, taking history is not so broad, and some questions are excluded at the beginning. And then when there is time in an ambulance, I can obtain some other information if the person is conscious. But at the beginning of such a critical situation, there isn’t too much time to obtain information extensively.* (P1) |
| Interpreting information | Demonstrates understanding of a patient’s condition and its changes | *This was so that in the case of some change in heart rhythm, we could promptly respond to it.* (P6) |
|  | Makes a working diagnosis | *A really good paramedic or a good leader of the team should be able to make a working diagnosis.* (P6) |
|  | Generates and considers an adequate number of different diagnoses | *The more the situation is different from routine ones, the greater the stream of thoughts, the more different diagnoses I think about, or how I would say that. In short, I try to consider more alternatives that could be taken into consideration compared to a routine case in which I’m more or less clear about the clinical picture.* (P19) |
|  | Identifies and resolves inconsistencies | *We didn’t know, but the set of injuries that I found out about by primary, secondary examination suggested . . . I said that he had had to have fallen from a greater height, that it couldn’t have been from that two metres. So, then we were figuring out where he had actually fallen from and what might have been a mechanism of that injury.* (P14) |
|  | Communicates interpretations regarding a patient’s condition and diagnosis to team members | *I think aloud very often. I think that also in this situation that I’ve described, I was thinking aloud about what it could be, what I was seeing on the ECG, so my colleagues could understand why I was doing that, why I was calling the cardio centre and so on. Because if I hadn’t announced that, then there would have been a misunderstanding.* (P13) |
|  | Discusses with or considers suggestions from team members regarding a patient’s condition and diagnosis | *When I have doubts, it is appropriate to ask the crew members for an opinion, because sometimes even a driver, when he sees similar cases ten times, can give an accurate suggestion of what the case could be about.* (P9) |
| Anticipating states | Anticipates a possible course of events | *According to me, to anticipate is needed in every situation. As I mentioned, the fact that the leader anticipates, that’s fine.* (P3) |
|  | Prepares for a possible negative course of events with alternative plans | *I’m trying to get ready for the worse situation when the patient is in a really poor condition. Simply put, to think about whether I’ll have to do this intervention and what if it is not effective or if I screw it up, what I’ll do. It has worked out best for me to think about plan B or maybe also plan C, just to think ahead.* (P2) |
|  | Takes action for the sake of a possible negative course of events | *When a patient is in a poor condition, I anticipate that circulatory arrest may occur, respiratory arrest may occur, briefly, that the condition can get worse rapidly. So, I have to anticipate that and prevent it getting worse.* (P4) |
|  | Communicates a possible negative course of events to team members | *A good leader anticipates and shares his/her anticipations regarding a course of events, because he/she sensitizes his/her co-workers in a team to notice whether the situation is really evolving in this way or not, and they can notice it earlier because the leader can pay attention to something else. At the same time, they can be prepared for that and they won’t be surprised.* (P8) |
|  | Discusses with or considers suggestions from team members regarding a possible negative course of events | *I just asked the colleague what he was thinking, if it could be that the patient might start to fibrillate or what. And he actually said that it could be that way. As well, from moment to moment, it looked like that. So, we both approved that and we were prepared for this alternative too.* (P18) |
|  | Involves team members in anticipation-related activities | *I would tell my colleague, for instance, to prevent this or this, we do this or we administer this, or we sit him down like this.* (P20) |
| Identifying options | Generates and considers an adequate number of alternative solutions | *We considered that if the air ambulance wasn’t available within an adequate time horizon, we would choose transportation by ground.* (P5) |
|  | Discusses with or considers suggestions from team members regarding solutions | *Usually, a good leader consults with the others on whether the administration of this drug is a good choice to prevent some complications or on the dosage of this drug. Or the transport, whether it is good to transport the patient there or there because he or she has such an injury, whether it is better to go there or there, or what is the best for the patient.* (P7) |
|  | Considers advantages and disadvantages of solutions | *A good leader has to find the middle way between enough invasive things that have benefits for the patient, but not to do unnecessary invasive things. Simply, to balance between what is still for the benefit of the patient and what is not. And that decision is sometimes tough.* (P16) |
|  | Evaluates relevant factors and is not affected by irrelevant factors | *In a common situation, you are not limited for time. In challenging situations, it’s about time. You are limited for time, you are under time pressure, you have to act quickly.* (P3) |
|  | Seeks input on various situation-related issues with relevant parties | *We have an application that connects us to the National Institute for Cardiovascular Diseases. We used it. From there, a clinician—a specialist—called and told us precisely what we should administer to the patient, besides what we usually administer.* (P15) |
|  | Involves team members in seeking input with relevant parties | *At my command. But I don’t need to tell him [the colleague], because it is clear. I tell him “Look for the ICU.” And he calls the dispatch centre and says “We have a patient who’s been resuscitated, we need the ICU.”* (P12) |
|  | Identifies leverage points and uses them in devising novel solutions | *A good paramedic can deal with every single situation when he hasn’t [equipment] for something . . . Improvise. Exactly. Every good and proper paramedic can also improvise.* (P14) |
| Implementing decisions | Implements a solution while gathering information | *The treatment has to go alongside diagnosing. Because in a common case, you are talking with the patient and then you examine him. You have relatively a lot of time. In resuscitation, it is not like that. The examination is ongoing with the resuscitation.* (P12) |
|  | Prepares tasks before implementing | *Meanwhile, I prepared the drugs that I can administer during the CPR, such as adrenaline.* (P1) |
|  | Tailors workspace and bystanders’ behaviour | *At that moment, we quickly relocated the patient because she was really in such a place where two couldn’t operate. So we put her into a more open space.* (P6) |
|  | Involves team members and other relevant resources in solving a situation | *The basis is that the leader must communicate mainly in such situations when it is important to give clear orders. There are also firemen in traffic accidents, but they are also just a part of the rescue system and they also sometimes need to know that somebody leads. And I say “Come with me, we’re going here,” “You, take him out,” “You, do this.”* (P10) |
|  | Communicates and explains decisions and actions to team members, other professionals, patients, and their close ones | *I mostly also explain to the patient in layman’s terms what is with him or her, why we are taking or not taking him or her, or what is needed, what to focus on when there is some chronic pain. So I always try to explain.* (P20) |
|  | Provides adequate situation reports | *Plus, it is good to contact the dispatch centre after the arrival and hand over the so-called situational report. It means to inform what happened there, for example, what risks there are, what the number of casualties is, what the injuries are.* (P5) |
|  | Implements a comprehensive solution systematically | *Maybe the most important thing is to have a system, not to panic and not to do things chaotically.* (P11) |
|  | Adapts implementing a solution to relevant and priority actions in a situation | *There can be a very mild allergic reaction, and there can be such a reaction that the patient is choking under your hands. But you don’t know that until you arrive on a scene. So, you modify the procedure according to that.* (P12) |
| Re-evaluating decisions | Re-assesses a patient | *He or she repeatedly re-assesses, measures vital functions, repeatedly returns to the ABCDE approach. I think a good leader re-assesses whether the pressure is good, how the heart action is, measures the pulse repeatedly, finds out by palpation whether it is tangible also on the wrist, not only in the carotids. He or she asks the patient how he or she is feeling, whether it relieves the pain . . .* (P4) |
|  | Adapts re-assessing a patient to relevant and priority information | *A repeated, more detailed re-assessment would be a disadvantage for the patient in the given situation.* (P9) |
|  | Allows adequate time for intervention to take effect | *I administer him the drug and I’m waiting to see whether it will stabilize. As I said, it rarely happens that he’s getting better. Usually, he only stabilizes. So I’m waiting to see whether the drug helps, whether it’s getting him to stabilize.* (P7) |
|  | Revises a solution in light of new information | *Considering that the problem had occurred again, that she had felt the chest pain again, that she had had the feeling of breath insufficiency again . . . so I changed the decision by admitting that it could be an acute coronary syndrome evolving and that it was necessary to admit her urgently, where other specialized examinations could be performed.* (P16) |
|  | Searches for more information and other options | *When the patient is not getting better, I think about why he is not getting better and what I’m going to do next. So, I perform another step. Then, when he is not getting better, I will perform another. It means to pay attention to the problem until I solve it.* (P2) |
|  | Involves team members in re-assessing a patient | *I told my colleague, “Please, watch the pulse, measure by palpation on the wrist whether it is still felt.” Because when it is on the wrist, then you know that there is still some pulse.* (P7) |
| Maintaining standards | Follows established practice guidance when appropriate | *If somebody works in emergency services, he or she has to know the guidelines, has to follow them. I think in this situation, the decision process was much simplified by the existing guidelines that are set, unalterable, and have to be like that.* (P16) |
|  | Can justify when not following established practice guidance | *I can recall a situation in which I acted in a non-standard fashion, maybe outside the rules, but in my opinion it was justified, although then I had to explain some procedures.* (P9) |
|  | Leads team members to follow established practice guidance | *I had a colleague who was not a paramedic. And I had a problem guiding him in the situation. He could be guided, it wasn’t like he went against my decisions, but several times I had to tell him the procedure that was correct in resuscitation. We have the ABCDE approach that we follow.* (P4) |
|  | Uses relevant experience and knowledge | *One thing is what I have learnt and the other thing is what I have gained through praxis. The practical skills that are not written in books, so-called life experiences. They give you more than anything. But you have to be learnt because you can’t go without the theory.* (P10) |
|  | Shows professional behaviour | *A leader should greet people politely. Ideally, he or she politely introduces him or herself. And he or she should be kind towards the people, even when you think the case is unimportant. He or she should maintain professional courtesy.* (P12) |
|  | Engages in learning activities | *A leader still needs to educate themselves. To repeat the situations. What, for example? The procedures regarding resuscitation need to be repeated, consolidated. As well, ways of treatment or some procedures can change. Or maybe such situations that do not happen so frequently, for example., some mass-casualty incidents.* (P11) |

*Note*. P1–20 = participant 1–20; CPR = cardiopulmonary resuscitation; ECG = electrocardiogram; ICU = intensive care unit.
